# Multi-Sonar Distributed Fusion for Target Detection and Tracking in Marine Environment

**DOI:** 10.3390/s22093335

**Published:** 2022-04-27

**Authors:** Roujie Chen, Tingting Li, Imran Memon, Yifang Shi, Ihsan Ullah, Sufyan Ali Memon

**Affiliations:** 1 School of Automation, Hangzhou Dianzi University, Hangzhou 310018, China; rexana3@163.com; 2 Science and Technology on Information Systems Engineering Laboratory, Nanjing 210007, China; orangeltt@163.com; 3 Department of Computer Science, Bahria University, Karachi Campus, Karachi 74200, Pakistan; imranmemon.bukc@bahria.edu.pk; 4 Department of Electrical and Computer Engineering, CUI Abbottabad Campus, Abbottabad 22060, Pakistan; ihsan@cuiatd.edu.pk; 5 Department of Defense Systems Engineering, Sejong University, Seoul 05006, Korea; sufyanahmedali@sejong.ac.kr

**Keywords:** out-of-sequence tracks, distributed fusion, track origin uncertainty

## Abstract

The multi-sonar distributed fusion system has been pervasively deployed to jointly detect and track marine targets. In the realistic scenario, the origin of locally transmitted tracks is uncertain due to clutter disturbance and the presence of multi-target. Moreover, attributed to the different sonar internal processing times and diverse communication delays between sonar and the fusion center, tracks unavoidably arrive in the fusion center with temporal out-of-sequence (OOS), both problems pose significant challenges to the fusion system. Under the distributed fusion framework with memory, this paper proposes a novel multiple forward prediction-integrated equivalent measurement fusion (MFP-IEMF) method, it fuses the multi-lag OOST with track origin uncertainty in an optimal manner and is capable to be implemented in both the synchronous and asynchronous multi-sonar tracks fusion system. Furthermore, a random central track initialization technique is also proposed to detect the randomly born marine target in time via quickly initiating and confirming true tracks. The numerical results show that the proposed algorithm achieves the same optimality as the existing OOS reprocessing method, and delivers substantially improved detection and tracking performance in terms of both ANCTT and estimation accuracy compared to the existing OOST discarding fusion method and the ANF-IFPFD method.

## 1. Introduction

The multi-sonar system is usually collaboratively deployed to realize the perception of the marine situation. Compared with the single sonar system, the multi-sonar system integrates multi-source information and can provide a much more comprehensive and accurate as well as reliable perception image of the marine environment. It has been widely applied in civil and military fields, such as underwater mapping [1,2], fish behavior assessment [3], marine life tracking [4], ship salvage [5], underwater archaeology [6], and anti-submarine warfare [7,8,9]. Synthetic aperture radar is also widely applied in marine traffic control [10], shipwreck rescue [11], and fishery management [12], which is a leading technique with the day and all-weather working capacity [13]. Due to the radar emitting electromagnetic signals, which are easily absorbed by seawater, it is suitable to detect the targets in the sea and is not conducive to the detection of underwater targets. In this paper, we focus on the multi-sonar fusion for jointly detection and tracking marine targets of interest which are under the seawater, for example, fish and submarines, in which, the tracks following targets can be timely initiated and then maintained, and also, the targets’ kinematic state such as position, velocity, etc., can be estimated in an accurate manner [14].

The multi-sonar fusion detection system can be roughly divided into the centralized and distributed [15,16]. In the centralized fusion architecture, each sonar directly transmits its returned raw measurements to the fusion center where these measurements are used for optimal estimation of the target kinematic state and the target existence state, while at the cost of tremendous communication and computation consumption, as well as the possible single-node failure problem. However, in the distributed fusion architecture, each sonar firstly operates the local tracking based on its raw measurements and only transmits tracks with high quality to the fusion center for subsequent track-to-track fusion, which means estimating the target kinematic state and the target existence state by local tracks [17]. Compared with the centralized fusion system, the distributed fusion system is able to deliver comparative fusion performance but with greatly reduced communication and computation consumption, and also much-enhanced system stability (free from single-node failure problem) [18]. Therefore, to achieve a trade-off between the fusion performance and resources consumption, and system stability, the distributed fusion framework is preferably adopted to implement the multi-sonar target detection and tracking in the marine environment [19]. Due to imperfect sensing, the origin of data returned by each sonar is usually uncertain, the data may originate either from targets of interest or clutter, and this problem is even exacerbated by the presence of multi-target and target misdetection. In addition, due to the different sonar internal processing times and diverse communication delays between sonar and the fusion center, the data measured at an earlier time usually arrives at the fusion center later than those measured at a later time, which causes chaos in the sequence of data arriving in the fusion center and becomes temporal out-of-sequence (OOS) in the fusion center. As a consequence, fusing those origin-unknown OOS data to improve multi-sonar detection and tracking performance becomes critically important. The most straightforward processing method is out-of-sequence discarding (OOS-D), that is, ignoring OOS data [20]. However, when the OOS data occurs frequently or comes from a sonar with higher measurement accuracy, the detection and tracking performance of the fusion center is significantly degraded due to directly discarding targets data. Therefore, specific methods need to be designed to fuse multi-sonar origin-unknown OOS data in a more efficient manner. According to the types of data received from local sonars, the multi-sonar OOS data fusion can be categorized into multi-sonar out-of-sequence measurement (OOSM) fusion and multi-sonar out-of-sequence track (OOST) fusion. In the OOSM fusion, the measurement of sonar arrives at the fusion center in chaos and is fused with the central track. In the OOST fusion, the fusion center receives the local track of sonar out of sequence and fuses it with the central track.

The OOSM fusion arises in the centralized fusion framework, in which each local sonar directly transmits its origin-unknown raw measurements to the fusion center with arriving OOS [21]. The multi-sonar OOSM fusion problem has been pervasively investigated and numerous approaches are proposed in the past decades. According to the efficiency of the algorithm, the OOSM fusion method can be divided into two categories which include the OOSM filter method and the OOSM association method. The OOSM filter method is described in detail mainly in references [22,23,24,25,26] and the OOSM association method is introduced in references [26,27]. Ref. [22] firstly proposed a retrodiction-based A1 algorithm for one-lag OOSM fusion in an optimal manner, and also provided a suboptimal but computationally more effective algorithm B1, which is developed by ignoring the retrodicted process noise fully accounted for in A1. The authors in [23] extended the single-lag specified B1 algorithm proposed in [18] to fuse multi-lag OOSM by processing the previously stored l-lag measurements in an iterative manner and proposed the Bl algorithm. To reduce the storage and computation consumption of Bl, the Bl1 algorithm was proposed in [24] and achieved the one-step fusion of multi-lag OOSM based on the defined equivalent measurement. Ref. [25] proposed a non-retrodiction-based fusion algorithm and employed the forward-prediction fusion and decorrelation to integrate the multi-lag OOSM, but suffered from fusion performance degradation when the lags of OOSM increase. In [26], the target’s historical and current states were augmented to fuse the multi-lag OOSM in an optimal Bayesian framework, and the augmented state Kalman filter (AS-KF) was proposed there without calculating the complicated cross-covariance between OOSM and the current state. The literature reviewed above ideally assumes perfect OOSM-to-track association and does not consider the data association and track management caused by the OOSM origin uncertainty. The authors in [26] combined the Probabilistic Data Association algorithm with the AS-KF to fuse the origin-unknown multi-lag OOSM in an optimal manner and also provided a suboptimal but computationally more effective solution therein. Recently, a distributed integrated PDA-forward prediction fusion and decorrelation (DIPDA-FPFD) method was proposed in [27] to efficiently fuse origin-unknown multi-lag OOSM in the multiple asynchronous bearing-only sensors tracking systems, and utilized the recursively calculated probability of target existence as a track quality measure to implement track management, enabling confirming and maintaining true tracks following targets, as well as recognizing and deleting false track not following any targets.

Different from the OOSM fusion, the OOST fusion usually arises in the distributed fusion framework, in which the sonar local tracks arrive in the fusion center with temporal OOS, and the correlation between local tracks and central tracks needs to be carefully considered due to common prior information and process noise. Compared to the OOSM fusion, the multi-sonar OOST fusion problem has been less investigated in the open literature. However, to save communication resources, most realistic multi-sonar systems send track information instead of raw measurements to the fusion center, thus the multi-sonar OOST fusion becomes practically important. Most existing methods combine the OOSM fusion algorithms with the track decorrelation techniques to solve the multi-sonar OOST fusion problem. The authors in [28] proposed an optimal Bayesian solution involving a joint probability density of the current and past states to implicitly account for the correlation between the OOST with the current track and utilized the existing AS-KF to fuse the decorrelated OOST. Ref. [29] investigated the equivalent pseudo-measurement method to decorrelate the local track and the center track and used the existing OOSM fusion algorithm *Bl*1 to achieve suboptimal but computationally efficient fusion of OOST fusion of OOST. Under the same assumption, Ref. [30] completely ignored the cross-covariance between the sonar OOST and central tracks and directly deployed the existing A1, B1, and C1 algorithms to fuse multi-sonar OOST in a suboptimal way. In the practical multi-sonar detection and tracking system, in addition to the OOST fusion problem, the track origin uncertainty caused by the presence of clutter and multi-target also poses a significant challenge to the distributed fusion system. Ref. [31] proposed an approximate method under the Linear Gaussian by using an information matrix fusion to integrate the OOSTs with the central track. Ref. [32] proposed a method for OOST fusion that avoids a complex calculation of cross-correlation between the local track and central track, which has an advantage in the sense of implementation simplicity. Very recently, Ref. [33] investigated the fusing of OOSTs with track origin uncertainty in a distributed fusion setup and proposed a novel all neighbor fusion-integrated forward prediction fusion and decorrelation (ANF-IFPFD) method. The proposed ANF-IFPFD enumerates and probabilistically evaluates all feasible OOSTs-to-central tracks association events, fuses the central tracks with extracted information purely contributed by the local OOSTs through an information decorrelation process, and also utilizes the fuse probability of target existence to carry out the track management. It delivers promising fusion performance compared to the existing methods while suffering from degradation as the increasing lags of OOSTs. In this paper, we focus on the multi-sonar detection and tracking under the framework of distributed fusion with memory, and the motivations and contributions of this paper are summarized as follows:

1. Propose a novel multiple forward prediction-integrated equivalent measurement fusion (MFP-IEMF) algorithm to fuse the multi-lag OOSTs with track origin uncertainty in an optimal manner, which is applicable in both synchronous and asynchronous multi-sonar fusion systems.
2. Recursively calculate the probability of target existence as a track quality measure to detect marine targets.
3. Propose a random central track initialization technique to timely detect the randomly born marine target.

The rest of the paper is organized as: Section 2 describes the problem, Section 3 presents the multi-sonar multi-lag OOST fusion with the proposed MFP-IEMF, Section 4 introduces implementation considerations, Section 5 performs simulation verification and analyzes the results, followed by conclusions in Section 6.

## 2. Problem Statement

### 2.1. Target Model

The target kinematic state comprises a two-dimensional position and velocity component and is discretized at time $t_{k}$, i.e., $x_{k}={\left[x_{k},y_{k},{\dot{x}}_{k},{\dot{y}}_{k}\right]}^{T}$, which is assumed to be linear and described by

(1) $$x_{k}=F_{k,k-1}x_{k-1}+v_{k}$$

where $F_{k,k-1}$ is the state transition matrix from time $t_{k-1}$ to $t_{k}$ and described by

(2) $$F_{k,k-1}=\left[\begin{array}{cc} 1 & \Delta T_{k,k-1}\\ 0 & 1 \end{array}\right]⊗I_{2}$$

where $\Delta T_{k,k-1}$ is the time interval of two continuous scans, $I_{2}$ is the 2D identity matrix. $v_{k}$ is the white process noise, which is represented as an additive Gaussian, with zero mean and covariance $Q_{k,k-1}$,

(3) $$Q_{k,k-1}=q\left[\begin{array}{cc} \frac{\Delta T_{k,k-1}^{3}}{3} & \frac{\Delta T_{k,k-1}^{2}}{2}\\ \frac{\Delta T_{k,k-1}^{2}}{2} & \Delta T_{k,k-1} \end{array}\right]⊗I_{2}$$

where q denotes the power spectral density with a constant value, which is a design parameter for the estimation filter and represents the uncertainty in the target model.

Due to the marine target stochastically disappearing and appearing at any time, the existence of the target is a stochastic event. Denoting the target existence and non-existence event at time $t_{k}$ by $\chi_{k}$ and ${\bar{\chi }}_{k}$, their temporal propagation process follows a first-order Markov chain and is mathematically described by

(4) $$\left[\begin{array}{c} P\left(\chi_{k}|Z^{k-1}\right)\\ P\left({\bar{\chi }}_{k}|Z^{k-1}\right) \end{array}\right]=M_{1}\left[\begin{array}{c} P\left(\chi_{k-1}|Z^{k-1}\right)\\ P\left({\bar{\chi }}_{k-1}|Z^{k-1}\right) \end{array}\right]$$

where $M_{1}$ is Markov state transition matrix and given as

(5) $$M_{1}=\left[\begin{array}{cc} P\left(\chi_{k}|\chi_{k-1}\right) & P\left(\chi_{k}|{\bar{\chi }}_{k-1}\right)\\ P\left({\bar{\chi }}_{k}|\chi_{k-1}\right) & P\left({\bar{\chi }}_{k}|{\bar{\chi }}_{k-1}\right) \end{array}\right]$$

where $P\left(\chi_{k}|\chi_{k-1}\right)$ is the probability that target exists at time $t_{k}$ given it did exist at time $t_{k-1}$, $P\left({\bar{\chi }}_{k}|\chi_{k-1}\right)$ is the probability that target exists at time $t_{k}$ given it did not exist at time $t_{k-1}$.

### 2.2. Sonar Model

At time $t_{k}$, each sonar η returns a set of measurements $Z_{k}\left(\eta \right)$ without prior knowledge of their origins, with η=1,2,…,s, s is the number of sonars deployed in the surveillance area. $z_{k}^{i}\left(\eta \right)$ denotes the ith measurement of $Z_{k}\left(\eta \right)$. By reason of imperfect detection, sonar returns the target measurement with a detection probability $P_{d}$. The sonar measures the target azimuth $\theta_{k}$ and range $r_{k}$ at time $t_{k}$, which is a nonlinear function of the sonar,

(6) $$z_{k}^{i}\left(\eta \right)=h\left(x_{k},z_{0}\left(\eta \right)\right)+w_{k}\left(\eta \right)=\left[\begin{array}{c} \sqrt{\left(x_{k}-x_{0}{\left(\eta \right)}^{2}+{\left(y_{k}-y_{0}\left(\eta \right)\right)}^{2}\right)}\\ \tan^{-1}\left(\frac{x_{k}-x_{0}\left(\eta \right)}{y_{k}-y_{0}\left(\eta \right)}\right) \end{array}\right]+\left[\begin{array}{c} r_{w,k}\left(\eta \right)\\ \theta_{w,k}\left(\eta \right) \end{array}\right]$$

where $z_{0}\left(\eta \right)={\left[x_{0}\left(\eta \right),y_{0}\left(\eta \right)\right]}^{T}$ is the sonar η position, $w_{k}\left(\eta \right)$ is the measurement noise, which is modeled by the Gaussian noise, with zero mean and covariance $R_{k}\left(\eta \right)$.

Except for possible target detection, sonar returned measurements $Z_{k}\left(\eta \right)$ also contain a random number of clutter measurements, with their number $m_{k}^{\eta }$ following a Poisson distribution

(7) $$\mu \left(m_{k}^{\eta }\right)=e^{-\rho V\left(\eta \right)}\frac{{\left(\rho V\left(\eta \right)\right)}^{m_{k}^{\eta }}}{m_{k}^{\eta }!}$$

where ρ is the clutter density, which can be set as estimated adaptively or known, which is usually assumed to satisfy a uniform distribution over the sonar measurement space V(η) and can be adaptively estimated or known.

### 2.3. Challenge

In the distributed fusion architecture with memory, each sonar η returns measurements $Z_{k}\left(\eta \right)$ by detecting marine targets with η=1,2,…,s at time $t_{k}$ and tracks the marine targets locally to obtain the local tracks ${\left\{p\left(x_{k}^{\zeta }\left(\eta \right),\chi_{k}^{\eta }\left(\eta \right)|Z^{k}\left(\eta \right)\right)\right\}}_{\zeta =1}^{M_{k}^{\eta }}$, where $M_{k}^{\eta }$ is the number of the local tracks in sonar η at time $t_{k}$ and $p\left(x_{k}^{\zeta }\left(\eta \right),\chi_{k}^{\zeta }\left(\eta \right)|Z^{k}\left(\eta \right)\right)$ denotes the updated hybrid state estimation of ζth local track in sonar η at time $t_{k}$,

(8) $$p\left(x_{k}^{\zeta }\left(\eta \right),\chi_{k}^{\zeta }\left(\eta \right)|Z^{k}\left(\eta \right)\right)=p\left(x_{k}^{\zeta }\left(\eta \right)|Z^{k}\left(\eta \right)\right)p\left(\chi_{k}^{\zeta }\left(\eta \right)|Z^{k}\left(\eta \right)\right).$$

where $p\left(x_{k}^{\zeta }\left(\eta \right)|Z^{k}\left(\eta \right)\right)$ is the posterior probability density function (pdf) of marine target kinematic state and $p\left(\chi_{k}^{\zeta }\left(\eta \right)|Z^{k}\left(\eta \right)\right)$ is the posterior probability of target existence (PTE). Once the PTE oversteps the confirmation threshold, the track is raised to a confirmed status. Then the set of local confirmed tracks $T^{k}\left(\eta \right)={\left\{p\left(x_{k}^{\zeta }\left(\eta \right),\chi_{k}^{\zeta }\left(\eta \right)|Z^{k}\left(\eta \right)\right)\right\}}_{\zeta =1}^{{\bar{M}}_{k}^{\eta }}$ is transmitted to the fusion center, where ${\bar{M}}_{k}^{\eta }$ denotes the number of the local confirmed tracks in the subset and ${\bar{M}}_{k}^{\eta }\leq M_{k}^{\eta }$. In ideal circumstances, the fusion center receives the local confirmed tracks $T^{k}={\left\{T^{k}\left(\eta \right)\right\}}_{\eta =1}^{s}$ from multiple sonars at time $t_{k}$ and updates the central track by fusing the hybrid state estimations of the central track at time $t_{k-1}$, i.e., $p\left(x_{k-1}^{f},\chi_{k-1}^{f}|T^{k-1}\right)$, with the local confirmed tracks $T^{k}$ at time $t_{k}$ to obtain the hybrid state estimation of the central track at time $t_{k}$, i.e., $p\left(x_{k}^{f},\chi_{k}^{f}|T^{k}\right)$. The fusion center obtains more accurate tracking and detection performance by combining the local tracks, including the estimation of kinematic state and the probability of target existence, from multiple sonars.

However, due to different communication delays between the sonar and the fusion center and the diverse sonar internal processing time, the fusion center receives data out of sequence. Aiming at the multi-lag OOST with memory fusion, the fusion center is required to solve three problems. Firstly, since the fusion center processes data in real-time, the OOSTs cannot be directly fused. Therefore, the fusion center is required to make reasonable use of OOSTs to improve the detection and tracking performances. Secondly, the local tracks and the central track have the same prior information and the same process noise that is caused by the measurements of all sonar being obtained by the same target model, so the fusion center ought to rule out the common information by using a decorrelation procedure. Thirdly, since the uncertain origin of the local tracks which are caused by multi-targets and clutter, the fusion center is required to initiate and maintain true tracks and delete false tracks.

**Figure 1:**
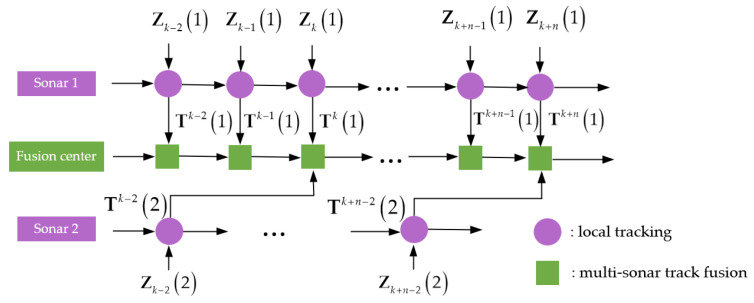
Multi-sonar OOSTs fusion framework.

Figure 1 shows an example with two sonars. The local tracks $T^{k-2}\left(2\right)$ arrive at the fusion center with two lags after the central tracks are updated at a later time $t_{k}$. Thus, these local tracks are OOSTs. The fusion center obtains the target hybrid state estimation of the central track, i.e., $p\left(x_{k}^{f},\chi_{k}^{f}|T^{k},T^{k-2}\left(2\right)\right)$, by using the OOS fusion method which fuses the OOSTs with the posterior pdf of the target hybrid state of the central track at time $t_{k}$, i.e., $p\left(x_{k}^{f},\chi_{k}^{f}|T^{k}\right)$. If the OOSTs are discarded, the center track is updated only by the data of sonar 1 which decreases the detection and tracking performances of the fusion center. At the same time, the local tracks $T^{k-2}\left(2\right)$ and the central track $p\left(x_{k}^{f},\chi_{k}^{f}|T^{k}\right)$ have the same process noise $v_{k-2}$ and the same prior information $T^{k-3}\left(2\right)$. The local tracks $T^{k-2}\left(2\right)$ include the true tracks of interesting targets and the false tracks. These problems of the correlation between the local track and the central track, the uncertain origin of the local tracks, and the local tracks arriving at the fusion center with OOS pose severe challenges to distributed OOS fusion with memory.

## 3. Multi-Sonar OOSTs Fusion with Track Origin Uncertainty

### 3.1. Framework of the Proposed MFP-IEMF Method

**Figure 2:**
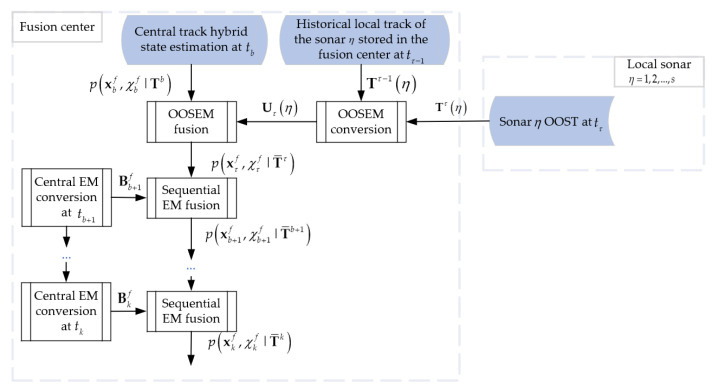
Flowchart of the proposed MFP-IEMF method.

Figure 2 describes the framework of the proposed MFP-IEMF by giving an example of fusing a central track f with a set of origin-unknow OOSTs $T_{\tau }\left(\eta \right)$ from sonar η formed up to time $t_{\tau }$. Firstly, the fusion center obtains the OOS equivalent measurements (OOSEMs) $U_{\tau }\left(\eta \right)={\left\{U_{\tau }^{\zeta }\left(\eta \right)\right\}}_{\zeta =1}^{{\bar{M}}_{\zeta }^{\eta }}$ by converting the OOSTs, aiming at ruling out the common information between the local track and the central track. Secondly, updating the hybrid state of the central track, i.e., $p\left(x_{\tau }^{f},\chi_{\tau }^{f}|{\bar{T}}^{\tau }\right)$, that is pre-stored at an earlier time $t_{b}$ by using the IEMF method to fuse the OOSEMs formed at the OOS time $t_{\tau }$. For the sake of consistency of notation, let $\left\{{\bar{T}}^{\tau }\right\}=\left\{T^{b},U_{\tau }\left(\eta \right)\right\}$. Thirdly, the central equivalent measurement at time $t_{b+1}$, i.e., $B_{b+1}^{f}$, is obtained by decorrelating between the central track estimation updated at time $t_{b+1}$ and the pre-stored central track estimation formed at time $t_{b}$. In the same way, let $\left\{{\bar{T}}^{\tau },B_{b+1}^{f}\right\}=\left\{{\bar{T}}^{b+1}\right\}$. Finally, updating the central track at time $t_{b+1}$, i.e., $p\left(x_{b+1}^{f},\chi_{b+1}^{f}|{\bar{T}}^{b+1}\right)$, by fusing the central equivalent measurement at time $t_{b+1}$ with the central track estimation at time $t_{b}$, and so on, through multiple forward predictions until the central track is updated to $p\left(x_{k}^{f},\chi_{k}^{f}|{\bar{T}}^{k}\right)$, where ${\bar{T}}^{k}=\left\{T^{b},U_{\tau }\left(\eta \right),{\left\{B_{i}^{f}\right\}}_{i=b+1}^{k}\right\}$.

In the realistic fusion system, multiple sonars measure the targets information in a synchronous or asynchronous manner. While the proposed MFP-IEMF method is capable of fusing both synchronous and asynchronous OOSTs, and their main fusion steps follow the framework of Figure 2, the details are slightly different. Therefore, synchronous and asynchronous OOSTs fusion will be introduced in detail, respectively, below.

### 3.2. Synchronous OOSTs Fusion

The MFP-IEMF consists of five steps. Synchronous OOSTs fusion is described in detail below.

#### 3.2.1. Out-Of-Sequence Equivalent Measurement Conversion

At time $t_{k}$, assume the fusion center receives a set of OOSTs $T^{\tau }={\left\{T^{\tau_{\eta }}\left(\eta \right)\right\}}_{\eta \in \left[1,s\right]}$ which are transmitted from different sensors and formed at different times. $T^{\tau_{\eta }}\left(\eta \right)={\left\{T^{\tau_{\eta },\zeta }\left(\eta \right)\right\}}_{\zeta =1}^{{\bar{M}}_{\zeta }^{\eta }}$ denotes the local confirmed tracks formed from sonar η at time $t_{\tau_{\eta }}$ and $T^{\tau_{\eta },\zeta }\left(\eta \right)$$t_{k}$ is the ζth track of $T^{\tau_{\eta }}\left(\eta \right)$. For each local track of the previous OOSTs’ set $T^{\tau -1}$, one obtains the predicted hybrid state pdf, i.e., $p\left(x_{\tau_{\eta }}^{\zeta }\left(\eta \right),\chi_{\tau_{\eta }}^{\zeta }\left(\eta \right)|Z^{\tau_{\eta }-1}\left(\eta \right)\right)$, by forward predicting its pre-stored hybrid state, with its mean and corresponded error covariance calculated by

(9) $$\begin{array}{l} {\hat{x}}_{\tau_{\eta }|\tau_{\eta }-1}^{\zeta }\left(\eta \right)=F_{\tau_{\eta },\tau_{\eta }-1}{\hat{x}}_{\tau_{\eta }-1|\tau_{\eta }-1}^{\zeta }\left(\eta \right)\\ P_{\tau_{\eta }|\tau_{\eta }-1}^{\zeta }\left(\eta \right)=F_{\tau_{\eta },\tau_{\eta }-1}P_{\tau_{\eta }-1|\tau_{\eta }-1}^{\zeta }\left(\eta \right)F_{\tau_{\eta },\tau_{\eta }-1}^{T}+Q_{\tau_{\eta },\tau_{\eta }-1} \end{array}$$

The ζth OOSEM of sonar η, without correlation with the central track, is obtained by decorrelating between the predicted hybrid state pdf and the posterior hybrid state pdf to rule out the common information between them, i.e., Uτηζ(η)=p(uτηζ(η)|Tτη,ζ(η),Tτη−1,ζ(η))=N(uτηζ(η);Eτηζ(η),Dτηζ(η)), with its mean and error covariance calculated via

(10) $$\begin{array}{l} D_{\tau_{\eta }}^{\zeta }\left(\eta \right)=H{\left({\left(P_{\tau_{\eta }|\tau_{\eta }}^{\zeta }\left(\eta \right)\right)}^{-1}-{\left(P_{\tau_{\eta }|\tau_{\eta }-1}^{\zeta }\left(\eta \right)\right)}^{-1}\right)}^{-1}H^{T}\\ E_{\tau_{\eta }}^{\zeta }\left(\eta \right)=H{\left({\left(P_{\tau_{\eta }|\tau_{\eta }}^{\zeta }\left(\eta \right)\right)}^{-1}-{\left(P_{\tau_{\eta }|\tau_{\eta }-1}^{\zeta }\left(\eta \right)\right)}^{-1}\right)}^{-1}\left({\left(P_{\tau_{\eta }|\tau_{\eta }}^{\zeta }\left(\eta \right)\right)}^{-1}{\hat{x}}_{\tau_{\eta }|\tau_{\eta }}^{\zeta }\left(\eta \right)-{\left(P_{\tau_{\eta }|\tau_{\eta }-1}^{\zeta }\left(\eta \right)\right)}^{-1}{\hat{x}}_{\tau_{\eta }|\tau_{\eta }-1}^{\zeta }\left(\eta \right)\right) \end{array}$$

Let $U_{\tau_{\eta }}\left(\eta \right)={\left\{U_{\tau_{\eta }}^{\zeta }\left(\eta \right)\right\}}_{\zeta =1}^{{\bar{M}}_{\tau_{\eta }}^{\eta }}$ denotes the set of OOSEMs of sonar η at time $t_{\tau_{\eta }}$, where ${\bar{M}}_{\tau_{\eta }}^{\eta }$ is the number of the OOSEMs in sonar η at time $t_{\tau_{\eta }}$.

The pseudo-function of the OOSEM conversion is described by

(11) $$\left[U_{\tau_{\eta }}^{\zeta }\left(\eta \right)\right]=EM\left(p\left(x_{\tau_{\eta }-1}^{\zeta }\left(\eta \right),\chi_{\tau_{\eta }-1}^{\zeta }\left(\eta \right)|Z^{\tau_{\eta }-1}\left(\eta \right)\right),p\left(x_{\tau_{\eta }}^{\zeta }\left(\eta \right),\chi_{\tau_{\eta }}^{\zeta }\left(\eta \right)|Z^{\tau_{\eta }}\left(\eta \right)\right)\right)$$

which contains Formulas (9) and (10).

#### 3.2.2. Data Sorting

Due to the fusion center receiving the local tracks of multiple sonars at different times, it is necessary to sort the OOSEMs according to the time sequence. Let $U_{\tau }={\left\{U_{\tau_{\eta }}\left(\eta \right)\right\}}_{\eta \in \left[1,s\right]}$ denote the OOSEM sorted in chronological order, where $\tau_{1}\leq \tau_{2}\ldots \leq \tau_{s}$.

#### 3.2.3. Fusion with OOSEMs

To fuse the central track f with the OOSEMs $U_{\tau }$, the pre-stored track hybrid state formed at time $t_{b}$ is updated forward to time $t_{\tau_{\eta }}$, where $t_{b}$ is equal to $t_{\tau_{\eta }}$. The resulted posterior hybrid state pdf of the central track f at time $t_{\tau_{\eta }}$ is calculated by

(12) $$f\left[p\left(x_{\tau_{\eta }}^{f},\chi_{\tau_{\eta }}^{f}|{\bar{T}}^{\tau_{\eta }}\right)\right]=IEMF\left(p\left(x_{b}^{f},\chi_{b}^{f}|T^{b}\right),U_{\tau_{\eta }}\left(\eta \right)\right)$$

where $p\left(x_{b}^{f},\chi_{b}^{f}|T^{b}\right)$ denotes the posterior hybrid state pdf of the central track f at time $t_{b}$, which is pre-stored in the fusion center.

The posterior pdf can be decomposed as

(13) $$p\left(x_{\tau_{\eta }}^{f},\chi_{\tau_{\eta }}^{f}|{\bar{T}}^{\tau_{\eta }},\right)=p\left(x_{\tau_{\eta }}^{f}|{\bar{T}}^{\tau_{\eta }}\right)p\left(\chi_{\tau_{\eta }}^{f}|{\bar{T}}^{\tau_{\eta }}\right)$$

Implementation steps of the IEMF method are introduced in detail next.

1. Gating technique

In order to reduce the computation burden, only a subset of OOSEMs $U_{\tau_{\eta }}\left(\eta \right)$ are chosen to update the central track f by using a gating technique. The gating technique is performed by calculating the Mahalanobis distance between the central track f and the OOSEM, and a subset of OOSEMs are chosen by

(14) $${\left(E_{\tau_{\eta }}^{\zeta }-{\hat{z}}_{b}^{f,\zeta }\right)}^{T}{\left(S_{b}^{f,\zeta }\right)}^{-1}\left(E_{\tau_{\eta }}^{\zeta }-{\hat{z}}_{b}^{f,\zeta }\right)\leq \gamma$$

where γ is the gating threshold, which is related to the gating probability $P_{w}$, ${\hat{z}}_{b}^{f,\zeta }$ and $S_{b}^{f,\zeta }$ are the mean and covariance of predicted measurement pdf $p\left(z_{\tau_{\eta }}^{f,\zeta }|{\bar{T}}^{\tau_{\eta }}\right)$, which are calculated by

(15) $$\begin{array}{l} {\hat{z}}_{b}^{f,\zeta }=H{\hat{x}}_{b|b}^{f}\\ S_{b}^{f,\zeta }=HP_{b|b}^{f}H^{T}+D_{\tau_{\eta }}^{\zeta }\left(\eta \right) \end{array}$$

Let ${\left\{U_{\tau_{\eta }}^{\zeta }\left(\eta \right)\right\}}_{\zeta =1}^{m_{\tau_{\eta }}^{\eta }}$ denote the subset of OOSEMs with cardinal numbers $m_{\tau_{\eta }}^{\eta }$ that are chosen, with $m_{\tau_{\eta }}^{\eta }\leq {\bar{M}}_{\tau_{\eta }}^{\eta }$.

2. Hybrid state estimation

The posterior PTE of the central track is calculated by

(16) $$P\left(\chi_{\tau_{\eta }}^{f}|{\bar{T}}^{\tau_{\eta }}\right)=\frac{1-\delta_{\tau_{\eta }}^{}\left(\eta \right)}{1-\delta_{\tau_{\eta }}^{}\left(\eta \right)P\left(\chi_{\tau_{\eta }}^{f}|T^{b}\right)}P\left(\chi_{\tau_{\eta }}^{f}|T^{b}\right)$$

where $\delta_{\tau_{\eta }}^{}\left(\eta \right)$ is the likelihood ratio and described by

(17) $$\delta_{\tau_{\eta }}^{}\left(\eta \right)=\{\begin{array}{cc} P_{d}\left(\eta \right)P_{w} & m_{\tau_{\eta }}^{\eta }=0\\ P_{d}\left(\eta \right)P_{w}-P_{d}\left(\eta \right)P_{w}\frac{V_{\tau_{\eta }}^{}\left(\eta \right)}{{\hat{m}}_{\tau_{\eta }}^{\eta }}\sum_{\zeta =1}^{m_{\tau_{\eta }}^{\eta }}p\left(z_{\tau_{\eta }}^{f,\zeta }|{\bar{T}}^{\tau_{\eta }}\right) & m_{\tau_{\eta }}^{\eta }>0 \end{array}$$

where $V_{\tau_{\eta }}\left(\eta \right)$ is the volume of the validation gate at time $\tau_{\eta }$ that is calculated by

(18) $$V_{\tau_{\eta }}\left(\eta \right)=\frac{\pi^{n_{z}/2}}{\left(n_{z}/2+1\right)!}\sqrt{\max{\left\{\left|S_{\tau_{\eta }}^{f,\zeta }\right|\right\}}_{\zeta =1}^{m_{\tau_{\eta }}^{\eta }}}\gamma^{\frac{n_{z}}{2}}$$

where $n_{z}$ is the degree of freedom and $m_{\tau_{\eta }}^{\eta }$ denotes the mean clutter number of sonar η at time $\tau_{\eta }$ which is obtained by

(19) $${\hat{m}}_{\tau_{\eta }}^{\eta }=\{\begin{array}{cc} 0 & m_{\tau_{\eta }}^{\eta }=0\\ m_{\tau_{\eta }}^{\eta }-P_{d}\left(\eta \right)P_{w}P\left(\chi_{\tau_{\eta }}^{f}|T^{b}\right) & m_{\tau_{\eta }}^{\eta }>0 \end{array}$$

The posterior pdf of the kinematic state is obtained by using the Gaussian mixture process, i.e.,  $p\left(x_{\tau_{\eta }}^{f}|{\bar{T}}^{\tau_{\eta }}\right)=N\left(x_{\tau_{\eta }}^{f};{\hat{x}}_{\tau_{\eta }|\tau_{\eta }}^{f},P_{\tau_{\eta }|\tau_{\eta }}^{f}\right)$, which is calculated via

(20) $$\begin{array}{l} {\hat{x}}_{\tau_{\eta }|\tau_{\eta }}^{f}=\sum_{\zeta =0}^{m_{\tau_{\eta }}^{\eta }}\beta_{\tau_{\eta }}^{\zeta }\left(\eta \right){\hat{x}}_{\tau_{\eta }|\tau_{\eta }}^{f,\zeta }\\ P_{\tau_{\eta }|\tau_{\eta }}^{f}=\sum_{\zeta =0}^{m_{\tau_{\eta }}^{\eta }}\beta_{\tau_{\eta }}^{\zeta }\left(\eta \right)\left(P_{\tau_{\eta }|\tau_{\eta }}^{f,\zeta }+\left({\hat{x}}_{\tau_{\eta }|\tau_{\eta }}^{f,\zeta }-{\hat{x}}_{\tau_{\eta }|\tau_{\eta }}^{f}\right){\left({\hat{x}}_{\tau_{\eta }|\tau_{\eta }}^{f,\zeta }-{\hat{x}}_{\tau_{\eta }|\tau_{\eta }}^{f}\right)}^{T}\right) \end{array}$$

where $\beta_{\tau_{\eta }}^{\zeta }\left(\eta \right)$ denotes the probability of equivalent measurement $U_{\tau_{\eta }}^{\zeta }\left(\eta \right)$ that originates from the target, which is obtained by

(21) $$\beta_{\tau_{\eta }}^{\zeta }\left(\eta \right)=\{\begin{array}{cc} \frac{1-P_{d}\left(\eta \right)P_{w}}{1-\delta_{\tau_{\eta }}\left(\eta \right)} & \zeta =0\\ \frac{P_{d}\left(\eta \right)P_{w}\frac{V_{\tau_{\eta }}^{}\left(\eta \right)}{{\hat{m}}_{\tau_{\eta }}^{\eta }}p\left(z_{\tau_{\eta }}^{f,\zeta }|{\bar{T}}^{\tau_{\eta }}\right)}{1-\delta_{\tau_{\eta }}\left(\eta \right)} & \zeta >0 \end{array}$$

${\hat{x}}_{\tau_{\eta }|\tau_{\eta }}^{f,\zeta }$ and $P_{\tau_{\eta }|\tau_{\eta }}^{f,\zeta }$ are the mean and error covariance of the kinematic state estimation updated using $U_{\tau_{\eta }}^{\zeta }\left(\eta \right)$ is calculated by

(22) $${\hat{x}}_{\tau_{\eta }|\tau_{\eta }}^{f,\zeta }=\{\begin{array}{cc} {\hat{x}}_{b|b}^{f} & \zeta =0\\ {\hat{x}}_{b|b}^{f_{\tau_{\eta }}\left({\hat{z}}_{b}^{f,\zeta }-E_{\tau_{\eta }}^{\zeta }\left(\eta \right)\right)} & \zeta >0 \end{array}|P_{\tau_{\eta }|\tau_{\eta }}^{f,\zeta }=\{\begin{array}{cc} P_{b|b}^{f} & \zeta =0\\ \left(I_{4}-K_{\tau_{\eta }}H\right)P_{b|b}^{f} & \zeta >0 \end{array}$$

where $K_{\tau_{\eta }}$ denotes the Kalman gain, which is described by

(23) $$K_{\tau_{\eta }}=P_{b|b}^{f}H^{T}{\left(S_{\tau_{\eta }}^{f,\zeta }\right)}^{-1}$$

#### 3.2.4. Central Equivalent Measurement Conversion

The central equivalent measurement of the central track f at time $t_{b+1}$ is obtained by decorrelating between the hybrid state posterior pdf of the central track f at time $t_{b+1}$ and at time $t_{b}$, i.e., $B_{b+1}^{f}$, which is calculated by

(24) $$\left[B_{b+1}^{f}\right]=EM\left(p\left(x_{b}^{f},\chi_{b}^{f}|T^{b}\right),p\left(x_{b+1}^{f},\chi_{b+1}^{f}|T^{b+1}\right)\right)$$

where $p\left(x_{b+1}^{f},\chi_{b+1}^{f}|T^{b+1}\right)$ denotes the posterior hybrid state pdf of the central track f at time $t_{b+1}$.

The mean and error covariance of $B_{b+1}^{f}$ are denoted $D_{b+1}^{f}$ and $E_{b+1}^{f}$.

#### 3.2.5. Sequential Fusion with Central Equivalent Measurement

The hybrid state posterior pdf of the central track f at time $t_{\tau_{\eta }}$ is fused with the central equivalent measurement $B_{b+1}^{f}$. The resulted hybrid state posterior pdf of the central track f at time $t_{b+1}$ is obtained by

(25) $$\left[p\left(x_{b+1}^{f},\chi_{b+1}^{f}|{\bar{T}}^{b+1}\right)\right]=IEMF\left(p\left(x_{\tau_{\eta }}^{f},\chi_{\tau_{\eta }}^{f}|{\bar{T}}^{\tau_{\eta }}\right),B_{b+1}^{f}\right)$$

It is worth noting that the detection probability $P_{d}^{f}$ of the fusion center needs to be carefully calculated by

(26) $$P_{d}^{f}=1-\prod_{\eta =1}^{s}\left(1-P_{d}\left(\eta \right)\right)$$

To update the hybrid state posterior pdf $p\left(x_{k}^{f},\chi_{k}^{f}|{\bar{T}}^{k}\right)$ of the central track f at time $t_{k}$ by iterating through three processes, Section 3.2.3, Section 3.2.4 and Section 3.2.5, when the set of OOSEMs contains data formed at multiple times.

### 3.3. Asynchronous OOSTs Fusion

Due to all sonar sampling times being exactly the same in the synchronous sampling system, in Equation (15), the posterior hybrid state pdf of the central track is obtained by straightforward fusing the OOSTs with the pre-stored hybrid state of the central track at the OOS time. In the asynchronous sampling system, the sampling time of the central track is not the same as that of the local track. Therefore, the central track needs forward prediction processing, after that the OOSTs fusion method can be carried out. The OOSEM conversion of the asynchronous sampling system is calculated in the same way as Equation (11). Due to the central track does not have the posterior pdf updated at time $t_{\tau_{\eta }}$, it forward predicts its pre-stored hybrid state pdf formed at time $t_{b}$ to time $t_{\tau_{\eta }}$. The predicted hybrid state pdf of the central track is calculated by

(27) $$p\left(x_{\tau_{\eta }}^{f},\chi_{\tau_{\eta }}^{f}|T^{b}\right)=p\left(x_{\tau_{\eta }}^{f}|T^{b}\right)p\left(\chi_{\tau_{\eta }}^{f}|T^{b}\right)$$

where $p\left(x_{\tau_{\eta }}^{f}|T^{b}\right)$ is the predicted pdf of target kinematic state, i.e., $p\left(x_{\tau_{\eta }}^{f}|T^{b}\right)=N\left(x_{\tau_{\eta }}^{f};{\hat{x}}_{\tau_{\eta }|b}^{f},P_{\tau_{\eta }|b}^{f}\right)$, with its mean and error covariance obtained by

(28) $$\begin{array}{l} {\hat{x}}_{\tau_{\eta }|b}^{f}=F_{\tau_{\eta },b}{\hat{x}}_{b|b}^{f}\\ P_{\tau_{\eta }|b}^{f}=F_{\tau_{\eta },b}P_{b|b}^{f}F_{\tau_{\eta },b}^{T}+Q_{\tau_{\eta },b} \end{array}$$

The predicted PTE $P\left(\chi_{\tau_{\eta }}^{f}|T^{b}\right)$ is calculated via

(29) $$P\left(\chi_{\tau_{\eta }}^{f}|T^{b}\right)=M_{1}P\left(\chi_{b}^{f}|T^{b}\right)$$

The posterior hybrid state pdf $p\left(x_{\tau_{\eta }}^{f},\chi_{\tau_{\eta }}^{f}|{\bar{T}}^{\tau_{\eta }}\right)$ is calculated in the same way as Equation (25). The remaining steps are the same as the OOSTs fusion in a synchronous sampling system.

## 4. Implementation Consideration

### 4.1. Marine Target Detection Strategy

In the realistic marine environment, the sonar returned measurement may either originate from targets of interest or clutter, and a huge number of tracks are initiated without prior knowledge on whether they are following the marine targets or not. In this paper, the recursively calculated PTE is utilized as a track quality measure to detect the marine targets in time, and also recognize and detect false tracks not following any targets.

The status of an initialized track is tentatively set to be unknown and subsequently updated based on its recursively calculated PTE. When the value of PTE exceeds the confirmation threshold, the track is raised to a confirmation status. Thus, it is deemed to follow a marine target of interest and remains at the to-be-confirmed status until its termination. Conversely, if the PTE falls below the termination threshold, the corresponding track is recognized as a false track not following any marine targets and is deleted from memory. Thanks to the proposed marine target detection strategy, the local sonar terminates the majority of false tracks, thus hugely saving the communication burden. At the same time, the fusion center can automatically detect the marine targets in time and maintain them effectively.

### 4.2. Random Central Track Initialization Technique

In the distributed fusion architecture with memory, the marine targets are randomly born and may be detected by part of the sonars in the system, which causes the fusion center to fail to observe the newborn targets in time and then greatly increase the probability of subsequent decision-making mistakes. Therefore, a random central track initialization technique is proposed to timely initiate and maintain the central track following the newborn targets, which initiates tracks by using the free equivalent measurements and operates fusion when the number of measurements within the gate is more than zeros.

**Figure 3:**
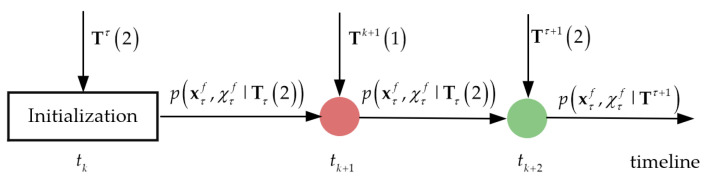
Fusion strategy framework of fusion center.

Taking two sonars as an example, it is assumed that the randomly born marine target A can only be detected through the OOS sonar 2. As shown in Figure 3, at time $t_{k}$, the fusion center receives a set of OOSTs $T^{\tau }\left(2\right)$. After fusion, the fusion center initializes a new track, i.e., $p\left(x_{\tau }^{f},\chi_{\tau }^{f}|T_{\tau }\left(\eta \right)\right)$, by using the free equivalent measurements that originate from the set of OOSTs. It is assumed that the mean and covariance of the free equivalent measurement are $E_{\tau }^{\zeta }\left(2\right)$ and $D_{\tau }^{\zeta }\left(2\right)$, respectively. The initial marine target kinematic state of the central track is obtained by the one-point initiation method, i.e.,

(30) $$\begin{array}{l} {\hat{x}}_{\tau |\tau }^{f}=\left[\begin{array}{c} E_{\tau }^{\zeta }\left(2\right)\\ 0\\ 0 \end{array}\right]\\ P_{\tau |\tau }^{f}=\left[\begin{array}{cc} D_{\tau }^{\zeta }\left(2\right) & 0_{2}\\ 0_{2} & \frac{v_{\max}^{2}}{\left(n+2\right)}\cdot I_{2} \end{array}\right] \end{array}$$

where $v_{\max}^{}$ denotes the maximum speed attainable by target, $0_{2}$ is a 2D zeros matrix. The PTE of the marine target is initialized to

(31) $$P\left(\chi_{\tau }|T^{\tau }\left(2\right)\right)=t_{ini}$$

where $t_{ini}$ is the initial parameter of the PTE.

The red circle in Figure 3 denotes that the gate fails and the track fusion is not performed and the green circle indicates that the gate is successful and the track fusion is performed. At time $t_{k+1}$, the fusion center receives the local tracks $T^{k+1}\left(1\right)$ uploaded by the sonar 1 that cannot observe target A, so the number of equivalent measurements within the gate is 0. Therefore, the central track is not processed. At this time, the hybrid state pdf of the central track is still $p\left(x_{\tau }^{f},\chi_{\tau }^{f}|T_{\tau }\left(2\right)\right)$. At time $t_{k+2}$, the fusion center receives the OOSTs $T^{\tau +1}\left(2\right)$ uploaded by the sonar 2 and the gate of the central track is successful. Thus, the hybrid state estimation $p\left(x_{\tau +1}^{f},\chi_{\tau +1}^{f}|T^{\tau +1}\right)$ is obtained, and the PTE of the central track increases. By comparison, the fusion center only processes the local tracks containing the data of targets, so as to ensure that the PTE does not decrease due to the unobservable part of the sonars, and eventually the fusion center cannot monitor the situation of randomly born marine targets. As a consequence, the random central track initialization technique proposed here is beneficial to speed up the detection of newly born marine targets, especially when they can be only observed by the OOS sonar.

## 5. Simulation Validation and Analysis

The novelty of the proposed multi-sonar distributed fusion method lies in three aspects: (1) the proposed MFP-IEMF is able to effectively fuse multi-lag OOSTs with track origin uncertainty and achieves optimal detection and tracking performance which delivers significant improvement over simply discarding the OOSTs; (2) the proposed method can be applied in both synchronous and asynchronous multi-sonar systems to improve the distributed fusion performance; (3) the MFP-IEMF enables the timely detection of the randomly born marine targets particularly when these targets can only be observed by the OOS sonars. To validate the novelty claimed above, three numerical experiments are carried out in the rest of the section.

### 5.1. Simulation Setup

**Figure 4:**
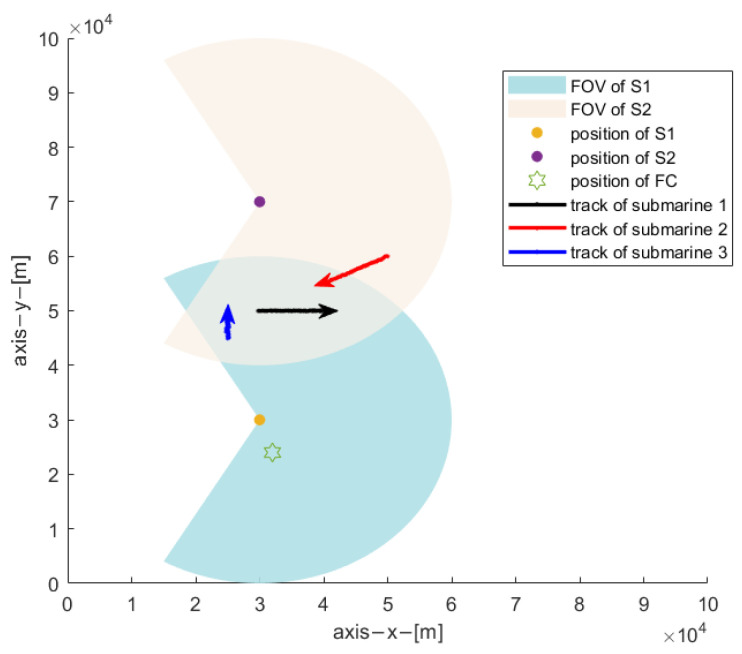
Simulation scenario, with FC denotes the fusion center.

A scenario of deploying the two-sonar system to detect and track three submarines is shown in Figure 4, two sonars are statically deployed at different positions to detect and track three moving submarines in two-dimensional space. Assuming that three submarines move at a nearly constant velocity model, their initial positions are: $x_{0}^{p}\left(1\right)={\left[30,50\right]}^{T}\mathrm{km}$, $x_{0}^{p}\left(2\right)={\left[45,60\right]}^{T}\mathrm{km}$, $x_{0}^{p}\left(3\right)={\left[25,45\right]}^{T}\mathrm{km}$; the initial speeds are $x_{0}^{v}\left(1\right)={\left[10,0\right]}^{T}m/s$, $x_{0}^{v}\left(3\right)={\left[-12,-6\right]}^{T}m/s$, $x_{0}^{v}\left(3\right)={\left[0,7\right]}^{T}m/s$, respectively. The submarines appear at different times, with submarine 1 appearing in the 1st second, submarine 2 appearing in the 200th second, and submarine 3 appearing in the 500th second.

**Figure 5:**
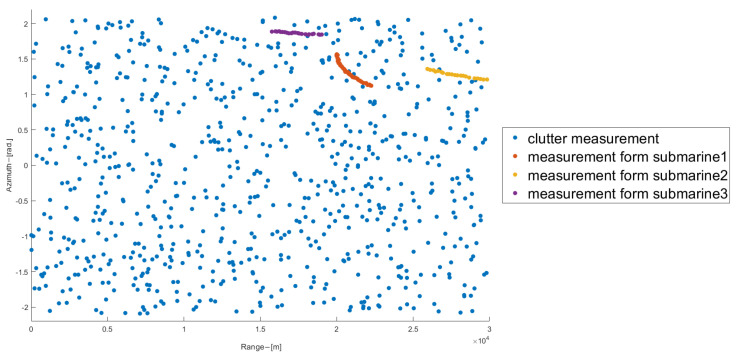
All measurements of sonar 1 within a single Monte Carlo experiment.

Suppose the sampling interval of each sonar is T=20 s, each experiment lasts 1000 s, the standard deviation of measurement noise is $\left[\Delta d,\Delta \theta \right]=\left[30m,0.3^{\circ }\right]$, and the number of Monte Carlo cycles is N=50. Each sonar owns a sector field of view V(η) described by its radius of 30 km and angle $\left[-120^{{}^{\circ}},120^{{}^{\circ}}\right]$. Both submarine 1 and 3 are commonly observed by the two sonars from the 1st to 1000th second, while, submarine 2 can only be observed by sonar 2 at the beginning period [1,541]s, and then is commonly observed by both sonars from the 541st second to 1000th second, in another, during [1,541]s that means the two-sonar system can never detect the submarine 2 if sonar 2 failed to transmit information related to submarine 2 to the fusion center. Owing to the complex marine environment, each sonar returns a set of origin-unknown measurements at each scan, including both the clutter measurements and target measurements, the target measurement is detected with a probability of 0.9, the number of clutter measurements at each scan is random and assumed to follow a Poisson distribution with mean value ${\bar{m}}_{k}^{\eta }$, with ${\bar{m}}_{k}^{\eta }=\rho \left(\eta \right)V\left(\eta \right)$, ρ(η) is the uniformly distributed clutter density. All measurements returned by sonar 1 accumulated over a single Monte Carlo experiment are shown in Figure 5, as can be seen there, submarines’ measurements are heavily contaminated by the clutter measurements. The sonar receives the raw measurements and performs local tracking, and transmits the confirmed local tracks to the fusion center for subsequent real-time distributed fusion. Since the communication distance between the sonar 1 and the fusion center is very close, its communication delay can be ignored, while the sonar 2 is far away from the fusion center, and its communication delay is large and cannot be ignored.

### 5.2. Simulation Results and Analysis

#### 5.2.1. Fundamental Case Study

The fusion center adopts the MFP-IEMF to fuse the multi-sonar OOSTs with track origin uncertainty in an optimal way to detect and track submarines automatically. In order to verify the superiority of the proposed method, this method is compared with the existing ANF-IFPFD method, OOS-D method, and OOS-Reprocessing (OOS-Re) method [34]. The ANF-IFPFD method is proposed to solve the problem of OOST fusion in a complex environment, which is a suboptimal fusion method. The OOS-D method simply discards the OOSTs and is served as the lower bound of multi-sonar OOSTs fusion to validate the improvement of the proposed method, while the OOS-Re method reprocesses all the OOSTs received since the last fusion in temporal sequence and delivers the optimal fusion performance but at the cost of intractable storage consumption and significant fusion delay, here serving as the upper bound.

**Experiment 1:** This experiment is used to verify the superiority of the proposed MFP-IEMF over existing methods in the synchronous sampling scenario. In this experiment, the sampling interval of the two sonars is T=20 s and both sonars start to measure in the 1st second. Therefore, it is a synchronous multi-sonar fusion system. Sonar 1 transmits the local tracks to the fusion center without delay, and sonar 2 transmits the local tracks to the fusion center with one lag.

The root mean square error (RMSE) and the averaged number of confirmed true tracks (ANCTTs) are designed to evaluate the detection and tracking performance of the algorithm. In order to give a fair comparison of the ANCTTs among compared methods, the track termination threshold, the track confirmation threshold, and the initial PTE are tuned to deliver the same number of confirmed false tracks (CFTs); it is 11 CFTs in this experiment.

**Figure 6:**
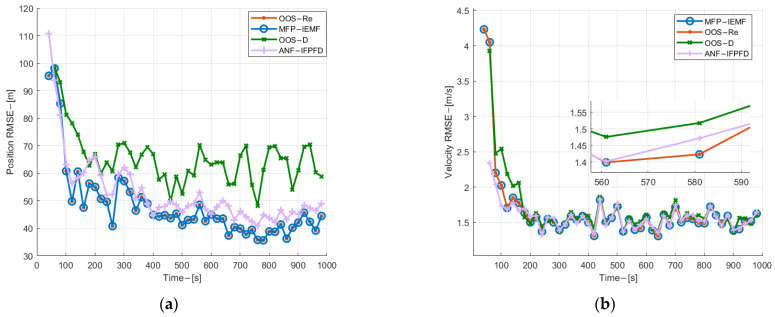

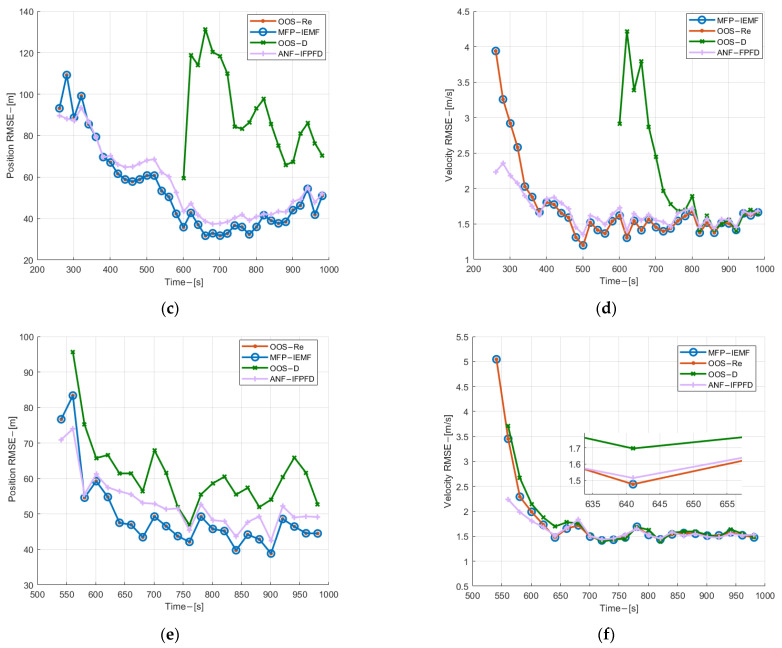
(**a**) Position estimation RMSE of submarine 1 in experiment 1; (**b**) velocity estimation RMSE for submarine 1 in experiment 1; (**c**) position estimation RMSE of submarine 2 in experiment 1; (**d**) velocity estimation RMSE for submarine 2 in experiment 1; (**e**) position estimation RMSE of submarine 3 in experiment 1; (**f**) velocity estimation RMSE for submarine 3 in experiment 1.

**Figure 7:**
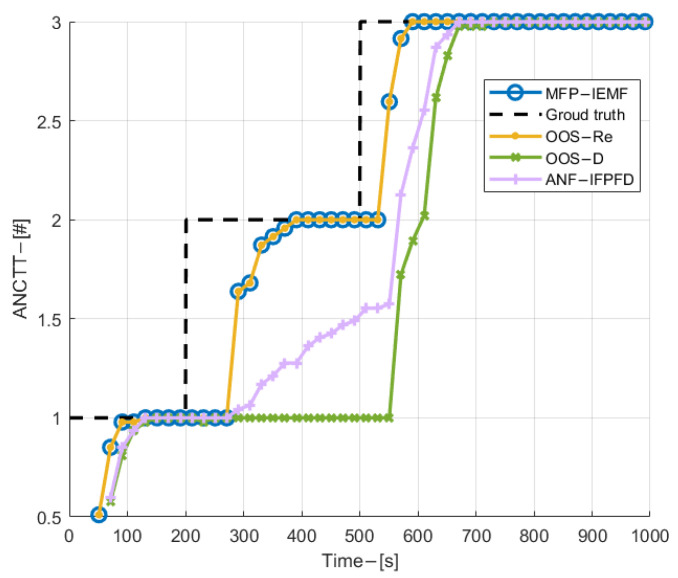
ANCTTs in experiment 1.

Figure 6 shows the velocity and position estimation RMSE for three targets with time among compared methods. As can be observed from Figure 6, in both velocity and position estimation, the MFP-IEMF method delivers obvious improvement compared with the OOS-D and the ANF-IFPFD after the central track converges. In addition, it gives the same performance as the OOS-Re in the synchronous sampling scenario. As shown in Figure 7, the proposed MFP-IEMF method initiates the CTTs faster compared with the ANF-IFPFD, the OOS-D, and as same as the OOS-Re. In particular, due to the random central track initialization technique, the MFP-IEMF quickly detects the appearance of submarine 2, while the OOS-D has about a 200 s time delay. In terms of track maintenance, the MFP-IEMF is also better than the OOS-D and has the same performance as the OOS-Re. Therefore, in detection and tracking performance, the MFP-IEMF is superior to the OOS-D and as optimal as the OOS-Re.

In addition to the detection performance and the tracking performance, the fusion time of each method in experiment 1 is compared in Table 1. All methods are implemented in MATLAB 2017b on the system with Intel(R) Core(TM) i7-10875H CPU, 2.30 GHz processor, 16 GB memory, and Windows 10 platform. The fusion time of each method is evaluated by its averaged elapsed computation time per experiment. As can be seen from Table 1, the average fusion time per experiment of the proposed MFP-IEMF with one lag is 11 s, much less than that of the OOS-Re method which on average requires more than 1000 s. In the case of little difference in fusion time, the OOS-D method and the ANF-IFPFD consume fusion time slightly less than the MFP-IEMF. It is obvious to conclude that the MFP-IEMF, ANF-IFPFD, and OOS-D methods are capable of fusion in real-time, but the OOS-Re method gives tracking results with delay and more fusion time is expected as the OOST lag increases. The reason that the OOS-Re method is required to reprocess all the local tracks to be in a chronological sequence, which causes tremendous fusion delay. In terms of storage requirements, the MFP-IEMF method only needs to store the set of central tracks’ information between the last OOS time and the next OOS time. However, the OOS-Re needs to store every set of local tracks received by the fusion center during two adjacent OOS times in addition to the set of central tracks’ information. Therefore, compared with the OOS-Re, the MFP-IEMF takes up less storage space.

**Experiment 2:** This experiment is used to verify the superiority of the proposed MFP-IEMF over existing methods in the asynchronous sampling scenario. In this experiment, the two sonars start sampling at different times, with sonar 1 in the 1st second and sonar 2 in the 2nd second. Other parameters are the same as experiment 1.

**Figure 8:**
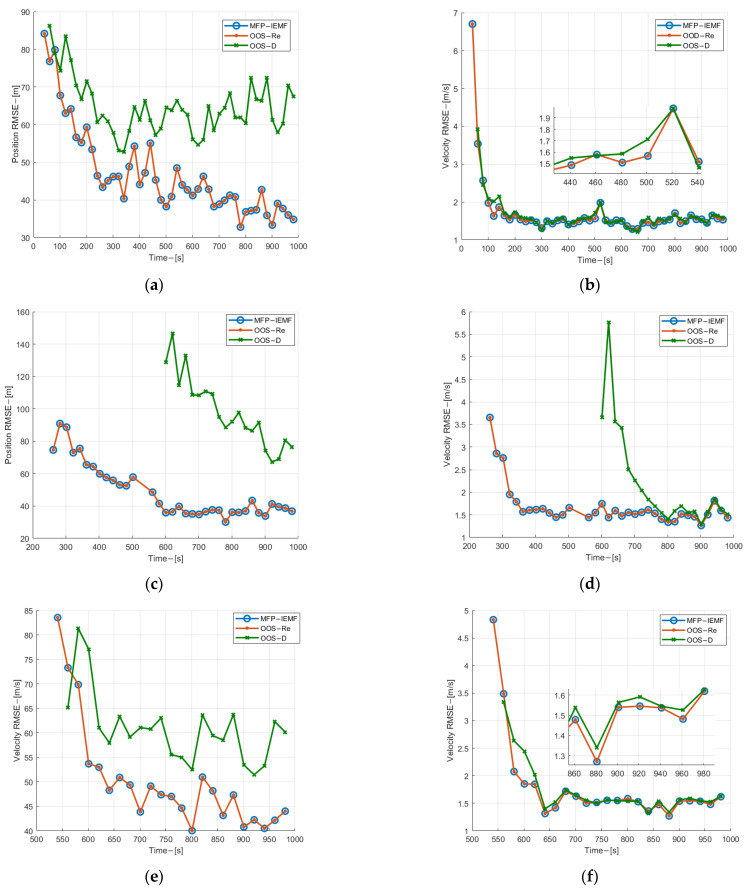
(**a**) Position estimation RMSE of submarine 1 in experiment 2; (**b**) velocity estimation RMSE for submarine 1 in experiment 2; (**c**) position estimation RMSE of submarine 2 in experiment 2; (**d**) velocity estimation RMSE for submarine 2 in experiment 2; (**e**) position estimation RMSE of submarine 3 in experiment 2; (**f**) velocity estimation RMSE for submarine 3 in experiment 2.

**Figure 9:**
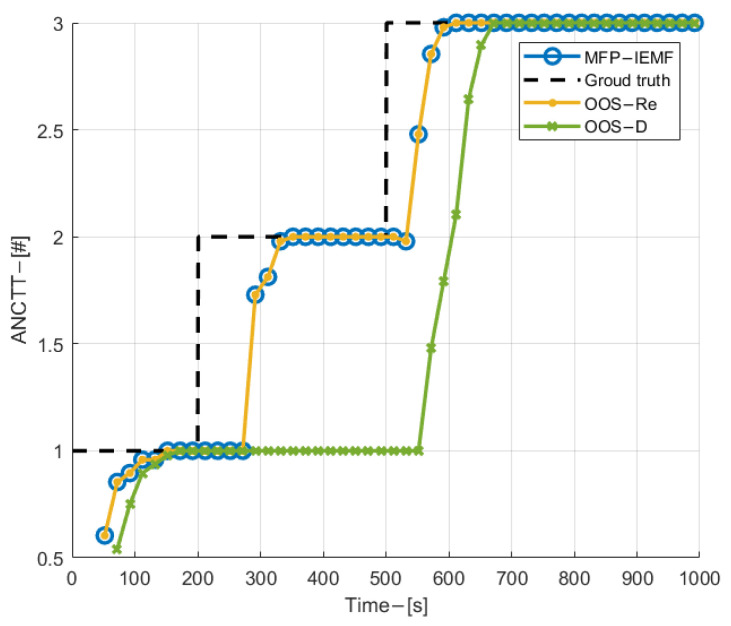
ANCTTs in experiment 2.

As can be seen from Figure 8, the MFP-IEMF has significant improvement in the velocity and position estimation RMSE compared with the OOS-D and has the same tracking performance as the OOS-Re. Different from ag 1, the curves of the MFP-IEMF and the OOS-D are farther away from each other in the track initialization phase for submarine 2 and they become closer and closer over time. The reason is that in the 600th second, the estimation error of the OOS-D is approximate to the measurement error, which decreases gradually through filtering, while the estimation error of the MFP-IEMF tends to be stable. Figure 9 shows the ANCTTs of the three methods, in which the track initialization of the MFP-IEMF and the track maintenance of the MFP-IEMF are better than that of the OOS-D and are the same as that of the OOS-Re. Thus, the MFP-IEMF is superior to the OOS-D and as optimal as the OOS-Re with detection and tracking performance in the asynchronous sampling scenario.

#### 5.2.2. Case Study on Different Delay Steps

This experiment is used to verify how the number of OOS lags affects the performance of the proposed MFP-IEMF. Sonar 2 is set to arrive at the fusion center in OOS with five lags and with thirty-five lags, respectively. Other simulation parameters are the same as experiment 1.

**Figure 10:**
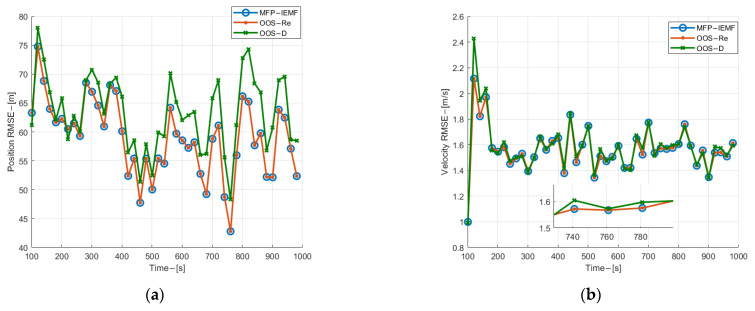
(**a**) Position estimation RMSE of submarine 1 with 5 lags; (**b**) velocity estimation RMSE for submarine 1 with 5 lags.

**Figure 11:**
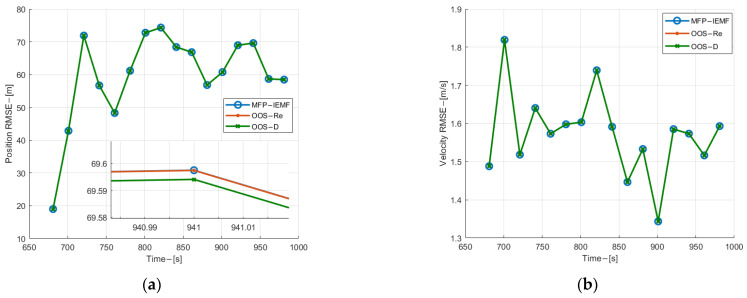
(**a**) Position estimation RMSE of submarine 1 with 35 lags; (**b**) velocity estimation RMSE for submarine 1 with 35 lags.

**Figure 12:**
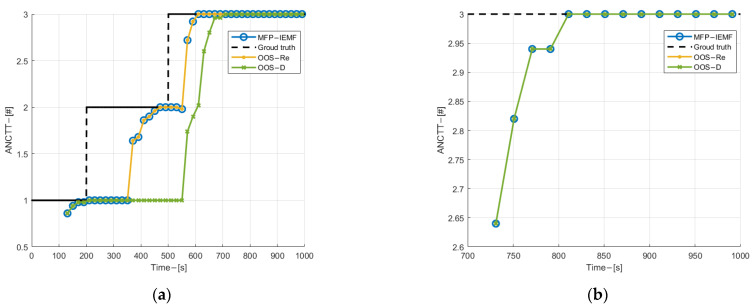
(**a**) ANCTTs with 5 lags; (**b**) ANCTTs with 35 lags.

Figure 10 and Figure 11 show the position and velocity estimation RMSE of submarine 1. As can be observed from Figure 10, when the OOS lags are five, the MFP-IEMF has an improvement in both velocity and position estimation compared with the OOS-D and the same performance with the OOS-Re. As shown in Figure 11, when the OOS lags are 35, the RMSE for all three methods is almost identical. Compared with the OOS-D, the MFP-IEMF has a subtle improvement. This is because the number of OOS lags is too large, and the OOSTs received by the fusion center are very small before the end of the experiment. As shown in Figure 12, when the OOS lags are five, the MFP-IEMF has improvement on both track initialization and track maintenance compared with the OOS-D and the same performance with the OOS-Re. However, when the OOS lags are 35, the ANCTTs of the MFP-IEMF do not have improved compared with the OOS-D. Due to a large number of lags, the OOSTs are received by the fusion center only after the true track of submarine 1 is successfully initialized with its PTE close to 1. In this case, the fusion of true track and OOSTs by the fusion center has no significant improvement in both detection and tracking performance as well as the PTE of the true track is less increasing.

## 6. Conclusions

This paper considers the multi-sonar information fusion for targets detection and tracking in the marine environment, and specifically investigates the multi-lag OOST fusion with track origin uncertainty under the distributed fusion framework with memory. The authors propose a novel multiple forward prediction-integrated equivalent measurement fusion (MFP-IEMF) method, which is capable of dealing with both synchronous and asynchronous multi-sonar tracks fusion problems. The proposed method fuses the multi-lag OOST with track origin uncertainty in an optimal manner and delivers substantially improved detection and tracking performance in terms of both ANCTT and estimation accuracy compared to the existing OOST discarding fusion method and the ANF-IFPFD method. Furthermore, a random central track initialization technique is also proposed by only using sonar measurements and few basic prior information, to detect the randomly born marine target in time via quickly initiating and confirming true tracks. The numerical results show that the proposed algorithm can be potentially implemented in the practical multi-sonar detection and tracking system. However, the proposed MFP-IEMF method is suitable for sparse multi-target scenarios with low target maneuvering. In the dense multi-target scenarios or target high maneuvering scenarios, the detection and tracking performance of the MFP-IEMF degrades. It is necessary to study the optimal fusion algorithm of OOST in a more complex environment.

## Figures and Tables

**Table 1:** Averaged fusion time of each compared method for experiment 1.

	MFP-IEMF	ANF-IFPFD	OOS-D	OOS-Re
Averaged fusion time per experiment (s)	11.7958	11.3148	10.8183	1014.2796
Real-time or delayed fusion	real-time	real-time	real-time	delayed
